# A Prospective Study of Tuberculosis Drug Susceptibility in Sabah, Malaysia, and an Algorithm for Management of Isoniazid Resistance

**DOI:** 10.1155/2015/261925

**Published:** 2015-03-09

**Authors:** Muhammad Redzwan S. Rashid Ali, Uma Parameswaran, Timothy William, Elspeth Bird, Christopher S. Wilkes, Wai Khew Lee, Tsin Wen Yeo, Nicholas M. Anstey, Anna P. Ralph

**Affiliations:** ^1^Department of Respiratory Medicine, Queen Elizabeth Hospital, 88000 Kota Kinabalu, Sabah, Malaysia; ^2^Infectious Diseases Unit, Clinical Research Centre, Queen Elizabeth Hospital, 88000 Kota Kinabalu, Sabah, Malaysia; ^3^Infectious Diseases Society Sabah-Menzies School of Health Research Clinical Research Unit, 88000 Kota Kinabalu, Sabah, Malaysia; ^4^Sabah Department of Health, 88000 Kota Kinabalu, Sabah, Malaysia; ^5^Luyang Outpatient Clinic, 88000 Kota Kinabalu, Sabah, Malaysia; ^6^Global and Tropical Health Division, Menzies School of Health Research, Darwin, NT 0811, Australia; ^7^Department of Medicine, Royal Darwin Hospital, Darwin, NT 0811, Australia

## Abstract

*Introduction*. The burden of tuberculosis is high in eastern Malaysia, and rates of *Mycobacterium tuberculosis* drug resistance are poorly defined. Our objectives were to determine *M. tuberculosis* susceptibility and document management after receipt of susceptibility results. *Methods*. Prospective study of adult outpatients with smear-positive pulmonary tuberculosis (PTB) in Sabah, Malaysia. Additionally, hospital clinicians accessed the reference laboratory for clinical purposes during the study. *Results*. 176 outpatients were enrolled; 173 provided sputum samples. Mycobacterial culture yielded *M. tuberculosis* in 159 (91.9%) and nontuberculous Mycobacterium (NTM) in three (1.7%). Among outpatients there were no instances of multidrug resistant *M. tuberculosis* (MDR-TB). Seven people (4.5%) had isoniazid resistance (INH-R); all were switched to an appropriate second-line regimen for varying durations (4.5–9 months). Median delay to commencement of the second-line regimen was 13 weeks. Among 15 inpatients with suspected TB, 2 had multidrug resistant TB (one extensively drug resistant), 2 had INH-R, and 4 had NTM. *Conclusions*. Current community rates of MDR-TB in Sabah are low. However, INH-resistance poses challenges, and NTM is an important differential diagnosis in this setting, where smear microscopy is the usual diagnostic modality. To address INH-R management issues in our setting, we propose an algorithm for the treatment of isoniazid-resistant PTB.

## 1. Introduction

Tuberculosis (TB) is a disease of major significance in eastern Malaysia (Malaysian Borneo), especially in Sabah state. Eastern Malaysia has a disproportionally high burden of the country's TB cases [1], and Sabah's estimated TB incidence rate (131/100,000) [2] far exceeds national rates (80/100,000) [3]. Rates of* Mycobacterium tuberculosis *drug resistance are not well defined in Sabah. Where testing has occurred, it has often been reserved for treatment failure cases, limiting the generalizability of such findings. Malaysian rates of multidrug resistant- (MDR-) TB have been estimated at 0.3% in 2005 and 1.3% in 2011 [4]. In unpublished data from the Sabah state reference laboratory, 16 MDR-TB cases occurred in 2011, representing 2.1% of isolates submitted that year [5]. Isoniazid resistance (INH-R) is reported in approximately 4% of* M. tuberculosis *in western Malaysia [6, 7], but rates have not been published from eastern Malaysia.

Isoniazid resistance (INH-R) is now generally acknowledged to be associated with poorer outcomes than INH-susceptible disease [8–11]. Despite INH-R TB being so common (4–10% globally [12–14]), the level of evidence to guide management is mostly level III-IV (Table 1), resulting in practices being nonuniform regarding drug choice, duration, and dosing frequency. The standard short-course regimen (isoniazid (H), rifampicin (R), ethambutol (E), and pyrazinamide (Z)) has traditionally been considered adequate [15, 16]. However 9 months of REZ (9REZ) or variations on this theme [10, 17–19] are increasingly used, and some advocate for the addition of a fluoroquinolone routinely [20], or in extensive INH-R pulmonary disease [18]. A new trial from the Tuberculosis Trials Consortium [21] provides much-needed evidence in this field; although the study was single-arm and nonrandomised, it provides evidence that twice or thrice weekly REZ for 6 months in HIV negative patients is mostly effective (3.5% failed or relapsed) but poorly tolerated [21]. Poor tolerability, especially of pyrazinamide, is a key challenge in the treatment of INH-R pulmonary TB (PTB) and a reason to require third-line options such as 12 months of a 2-drug regimen [22] or substitution with a fluoroquinolone [18].

The time taken to obtain* M. tuberculosis *susceptibility results is long if solid culture medium only is used, or where results are batched for dispatch to a reference laboratory; both these factors apply in our setting. If susceptibility results are delayed and the patient has already been switched to HR continuation phase treatment, the question of what to do after a period of effective monotherapy with rifampicin arises. Tailoring of regimens for pulmonary INH-R TB depending on factors, such as patient age, HIV status, and cavitary disease, can be difficult in high TB-burden settings accustomed to standardized regimens.

Knowledge of local susceptibility not only allows tailoring of treatment in individuals but provides local antibiogram data to aid clinicians in constructing regimens to use empirically and in culture-negative suspected tuberculosis (e.g., paediatric and extrapulmonary TB) and latent TB. Our first objective was to determine TB drug susceptibility profiles among patients with new or retreatment pulmonary TB presenting to a large community TB clinic in Kota Kinabalu, Sabah, Malaysia. Our second objective was to document clinical practice on receipt of susceptibility results.

## 2. Methods

This is a prospective observational study of smear-positive PTB outpatients, enrolled at Luyang Tuberculosis Outpatient Clinic, Kota Kinabalu, Malaysia. This is the main referral TB clinic in Kota Kinabalu, diagnosing and treating almost 200 patients annually. Kota Kinabalu district has a population of approximately 600,000. Participants were enrolled over 2 years, 4 July 2012–3 July 2014. Additionally, during the study period, clinicians at the Kota Kinabalu tertiary adult referral hospital, Queen Elizabeth Hospital (QEH), were able to access the services of the reference laboratory for clinical purposes, for patients in whom mycobacterial infection, particularly drug resistance, was suspected. QEH manages approximately 200 smear-positive PTB inpatients annually.

### 2.1. Informed Consent

Potentially eligible participants were provided with information about the study using written and pictorial materials and explanations from research staff and were required to provide written, informed consent. Consent was obtained from a parent/guardian for participants aged between 15 and 17, who additionally provided their assent.

### 2.2. Participants

Outpatient participants were eligible if they had sputum smear-positive pulmonary TB, were aged ≥15 years, and had received <7 days' TB treatment. They were referred for enrolment by the TB clinic doctor after being diagnosed with TB on the basis of clinical and X-ray assessment, and at least one sputum positive for acid fast bacilli (AFB) on Ziehl-Neelsen stain performed at the clinic's smear microscopy laboratory.

### 2.3. Procedures

A new sputum sample was provided on the day of enrolment. Samples were batched in a domestic refrigerator and transported to an international reference laboratory (Singapore General Hospital Laboratory, Singapore) as described elsewhere [27]. The BACTEC* Mycobacterium* Growth Indicator Tube (MGIT) 960 tube system was employed for culture. Drug susceptibility testing was performed for* M. tuberculosis* isolates using the nonradiometric MGIT system for isoniazid, rifampicin, ethambutol, streptomycin, and, in the instance of any first-line resistance, also for ofloxacin, kanamycin, and ethionamide. Nontuberculous mycobacteria (NTM) were identified using a DNA probe (ProbeTec, Becton-Dickinson). Further speciation, if done, was achieved using a second DNA probe (INNO-LiPA MYCOBACTERIA, Innogenetics, Ghent, Belgium) and high-performance liquid chromatography (HPLC) of mycolic acids.

HIV was tested using two rapid tests (Abbott Determine HIV-1/2 and a second locally supplied point-of-care test, as described elsewhere [28]). Positive results were confirmed by enzyme immunoassay, particle agglutination, and line immunoassay.

### 2.4. Ethical Approval

Ethical approval was obtained from the Medical Research Ethics Committee, Malaysian Ministry of Health, and the Health Research Ethics Committee of Human Research Ethics Committee of the Northern Territory Department of Health and Menzies School of Health Research, Australia.

### 2.5. Clinical Specimens from Hospital Patients

During the study period, clinicians at Queen Elizabeth Hospital were also able to utilise the arrangement established with the reference laboratory. Clinicians could send sputum specimens or extrapulmonary samples, with the batched research specimens, to obtain mycobacterial culture and susceptibility testing to guide clinical management. No specific inclusion criteria were applied apart from the suspicion of mycobacterial infection and difficulty obtaining results locally.

### 2.6. Statistics

Data were analysed using Stata 13.1. (Stata Corp., College Station, Texas, USA). Continuous variables were compared using Student's* t*-test or Wilcoxon Rank Sum test. Categorical variables were compared using Chi-squared or Fisher's Exact test, as appropriate.

## 3. Results

### 3.1. Outpatients

**Figure 1:**
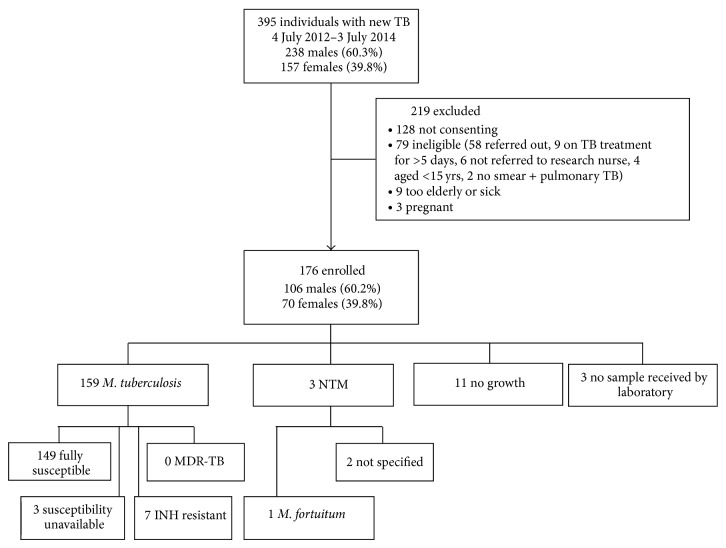
Outpatient study diagram showing enrolment and microbiological results.

During the 2-year study period, 176 outpatients consented and were enrolled (Figure 1, Table 2). Details relating to patient demographics and HIV status are described elsewhere [28]. A sputum sample was received by the laboratory for 173 individuals. Thirteen participants reported having received TB treatment in the past (median 12.5 years ago according to patients' recollection, range 1–39 years).

### 3.2. Outpatient Culture and Susceptibility Results

Mycobacterial culture was positive in 162 of 173 (93.6%) samples received. One hundred and fifty-nine (91.9%) yielded* M. tuberculosis*, and three (1.7%) yielded NTM; one was* M. fortuitum*; the others were not speciated. Of patients in whom NTM was isolated, two had underlying bronchiectasis.

Susceptibility results were unavailable in 3* M. tuberculosis *isolates due to overgrowth of the culture by contaminating organisms. In the 156 isolates from outpatients with susceptibility data, none had MDR-TB or rifampicin monoresistance. Seven people (4.5%) had infection with INH-R* M. tuberculosis*, none of whom recalled having previously received TB treatment. INH-R disease was more common in people of non-Malaysian ethnicity: 6 of the 7 (86%) were Filipino or Indonesian, compared with 50/159 (33.6%) with INH-S disease (*P* = 0.009).

One* M. tuberculosis* isolate with isoniazid resistance had additional streptomycin and ethionamide resistance. One instance of streptomycin monoresistance was also identified.

### 3.3. Management of Drug Resistant Cases

Among people identified as having INH-R, there were substantial delays between commencement of the first-line tuberculosis treatment regimen and change to a second-line regimen. The median delay was 13 weeks (range 8 to 28 weeks); see Table 3. Opportunities for delay included the time taken to dispatch sputum samples to the laboratory, receive the resistance report, inform the treating doctor, and recall the patient and for nursing staff to implement the doctor's new treatment regimen. The usual regimen prescribed after notification of INH-R results was rifampicin, pyrazinamide, and ethambutol (Table 3). Three patients with INH-R disease had follow-up sputum cultures performed at two months; all three had converted their culture to negative.

### 3.4. Hospital Patients

Fifteen additional samples were submitted for culture from hospital inpatients with suspected pulmonary TB (10 patients), or suspected extrapulmonary TB (five patients). Of those with suspected pulmonary TB, three had prior TB treatment failure, one had defaulted, three had unknown past treatment history, and three had underlying lung disease.

**Figure 2:**
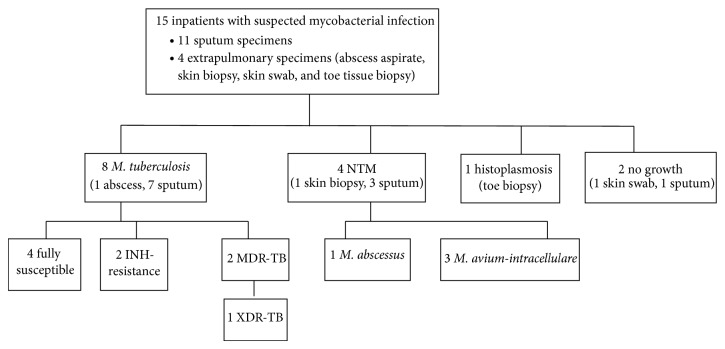
Hospital inpatients with suspected mycobacterial infection.

Findings from hospital inpatients are shown in Figure 2. Two cases of pulmonary MDR-TB, including one case of XDR-TB, were identified, both in heavily pretreated people. The XDR-TB case is the subject of a separate case report [29]. Two instances of INH-R were also identified, one monoresistant (in a pretreated patient) and one combined with streptomycin resistance (in a TB treatment-naïve health care worker). Regarding NTM, these were isolated in specimens obtained from four of the hospitalised individuals and identified by DNA probe and HPLC as* M. abscessus* or* M. avium-intracellulare* complex (from sputum) and* M. avium-intracellulare* complex (from a skin biopsy).

## 4. Discussion

We have found low rates of drug resistance among outpatient cases of tuberculosis in Sabah, Borneo, but occasional cases of MDR-TB in pretreated patients in the inpatient setting. The rate of isoniazid resistance (4.5%) is, reassuringly, at the lower end of what is reported globally [12–14]. Thus, while the TB burden is high in eastern Malaysia, our study illustrates that this appears to be predominantly drug-susceptible disease. While the uncommon finding of MDR-TB reflects well on the local TB control program, high ongoing rates of drug-susceptible disease in relatively young people (median age 30 years) suggest that community transmission of TB is an important area requiring targeted intervention.

The MDR-TB rates we have found are low compared with Malaysia's immediate neighbours, where rates have been estimated as follows in new and retreatment cases, respectively: Singapore 0.1% new and 2.9% retreatment, Thailand 1.7% and 34.5%, Indonesia 2% and 14.7%, and Philippines 4.0% and 20.9% [30]. There were few retreatment cases in our series (13 outpatients and 3 hospitalised patients) to provide an accurate assessment among these patients, but the two cases of MDR-TB both occurred in pretreated individuals. These low rates also compare favourably with other member states of the WHO Western Pacific Region; MDR-TB rates in Papua New Guinea, for instance, have been estimated to be at least 26% of cases [31].

**Figure 3:**
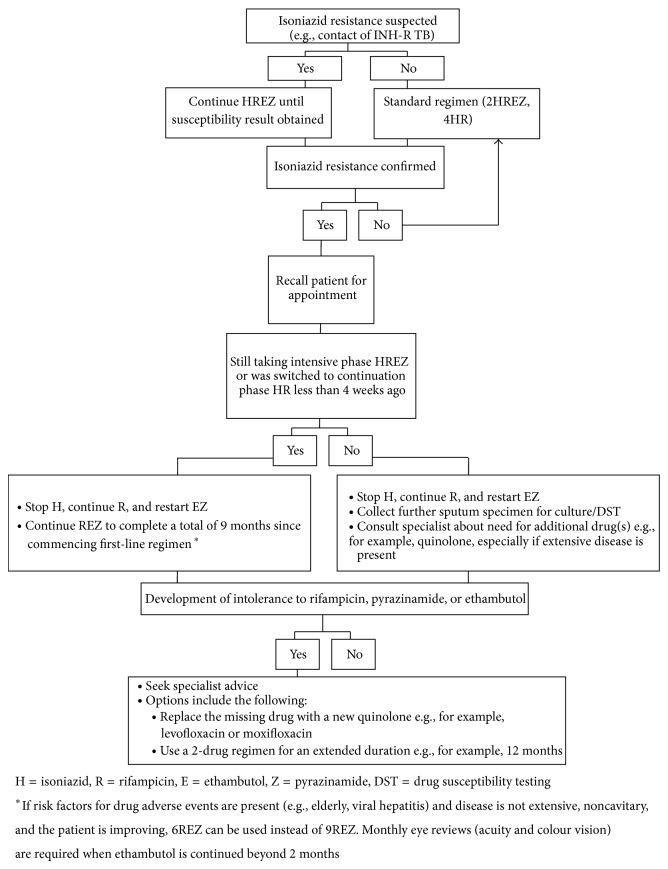
Algorithm for managing isoniazid resistance.

We identified substantial challenges in managing INH-resistance, particularly in the transition from protocol-based standardised care to tailored care. Despite Malaysian guidelines now recommending baseline cultures in all cases of suspected TB, routine susceptibility results are generally unavailable; usual practice in our setting is to undertake sputum mycobacterial cultures only for persistent smear-positivity after two to three months of treatment. Multifactorial delays occurred in implementing regimen changes, comprising the delay in specimens reaching the reference laboratory, the time taken to notify of the treating doctor, patient recall, and dispensing of the new regimen. A specific challenge in this context was that susceptibility results were frequently reported after completion of intensive phase treatment, when the routine switch to HR had already been made. Arising from these findings, we therefore developed an algorithm for managing this situation (Figure 3), based on the evidence summarised in Table 1. Such an algorithm does not replace clinical judgement guided by individual patient needs but provides guidance for nonspecialists in the local health centre settings where most outpatient TB is managed.

NTM was detected in 7 of 191 people (outpatients and inpatients) with suspected TB. This finding illustrates the problem of NTM colonisation or infection being mistaken for TB, especially where smear microscopy is the diagnostic mainstay. NTM infections have been noted to cause a substantial burden of disease in our clinical practice and elsewhere in Malaysia [32]. Rising rates have also been reported elsewhere in Asia [33, 34]. This disease has been relatively neglected compared with TB due to lower incidence and lack of associated public health risk, yet diagnosis and management can be far more challenging than TB and outcomes poor. More research is required in Sabah to better understand and optimise management of local NTM infections. Greater local access to mycobacterial culture is needed to aid clinicians in distinguishing NTM from TB, especially in patients in whom NTM is more likely, that is, those with underlying chronic lung disease.

A limitation of the study is that findings from the outpatient setting cannot be extrapolated to all TB cases in Sabah and are not representative of the inpatient setting; we have presented results from the selected group of hospital patients to illustrate this, but more data are required on inpatient drug resistance rates, as well as treatment outcomes.

In conclusion, current rates of MDR-TB in the community in Sabah are low, reflecting well on local TB case management, but INH-resistance poses challenges. This must be addressed to avoid additional stepwise acquired resistance. Improvement in local resourcing to ensure that diagnostics such as liquid mycobacterial culture or molecular tests are available in a timely, clinically useful fashion would be a major advance in TB management in Sabah, which has Malaysia's highest TB burden. In the current scenario of delayed access to susceptibility results, our proposed management algorithm can provide guidance here and in settings facing similar issues globally.

## Figures and Tables

**Table 1:** Treatment of isoniazid-resistant pulmonary tuberculosis: recommendations from selected references and level of evidence.

Treatment element	Reference	Study type	Level of evidence^*^
*Drugs *			
R throughout	[11]	Meta-analysis of trials and cohort studies	III
REZ	[21]	Nonrandomised trial	IIa
	[22]	Observational study	III
	[17, 23]	Expert opinion	IV
HREZ	[16]	Retrospective review	III
Add a fluoroquinolone in all instances (moxifloxacin 400 mg or levofloxacin 750–100 mg substituted for isoniazid)	[20]	Expert opinion	IV
Add a fluoroquinolone if pyrazinamide is not tolerated	[23]	Expert opinion	IV
Add a fluoroquinolone if disease is extensive	[18]	Expert opinion	IV
High-dose H is not recommended	[23]	Expert opinion	IV
*Duration *			
6 months (REZ)	[21]	Nonrandomised trial	IIa
6 months (REZ) if disease is not extensive	[23]	Expert opinion	IV
6 to 9 months (REZ)	[17]	Expert opinion	IV
6 to 12 months (variety of regimens)	[22]	Observational study	III
9 months (REZ) if culture-positive at 2 months	[23]	Expert opinion	IV
*Dosing frequency* ^**^			
Dosing should be daily during the intensive phase	[11]	Meta-analysis of trials and cohort studies	III
	[24]	Systematic review	
	[23]	Expert opinion	IV
Dosing can be given intermittently (thrice weekly) during the continuation phase	[25]	Expert opinion	IV
Twice or thrice weekly RZE for 6 months in HIV negative disease	[21]	Nonrandomised trial	IIa

^*^Ia: evidence from meta-analysis of randomized controlled trials, Ib: evidence from at least one randomized controlled trial, IIa: evidence from at least one well designed controlled trial which is not randomized, IIb: evidence from at least one well designed experimental trial, III: evidence from case, correlation, and comparative studies, IV: evidence from a panel of experts [26].

^**^Where dosing is self-administered rather than directly observed (as in Malaysia), there may be no advantage in an intermittent regimen.

**Table 2:** Characteristics of outpatients enrolled at Luyang Clinic.

Baseline characteristic	Number
Number	176
Age in years: median (range)	30.0 (16–73)
Male: number (%)	104 (59.0%)
Nationality	
Malaysian: number (%)	117 (66.5%)
Non-Malaysian: number (%)	59 (33.5%)
HIV positive: number (%)	3 (1.7%)
Current smoker	40 (22.7%)
Past TB	13 (7.4%)

**Table 3:** Isoniazid resistance among outpatients with pulmonary tuberculosis.

	Previous TB	Change in therapy after INH-R result	Time elapsed between treatment start & change of regimen	Second line regimen	Recommended duration of second line regimen	Outcome
Patient 1	No	Yes	28 weeks	RZE	6	Cured
Patient 2	No	Yes	15 weeks	RZE	9	Defaulted but already smear negative and received 6 months' treatment in total
Patient 3	No	Yes	8 weeks	RZE	4.5	Transferred
Patient 4	No	Yes	12 weeks	RZE	9	Cured
Patient 5	No	Yes	11 weeks	RZE	6	Cured
Patient 6	No	Yes	16 weeks	RZE	6	Cured
Patient 7	No	Yes	8 weeks	RZE	6	Cured
Median			**12 Weeks**			
